# Effectiveness of alpha-lipoic acid in patients with neuropathic pain associated with type I and type II diabetes mellitus: A systematic review and meta-analysis

**DOI:** 10.1097/MD.0000000000035368

**Published:** 2023-11-03

**Authors:** Mathias Orellana-Donoso, Michelle López-Chaparro, Marisol Barahona-Vásquez, Andrés Santana-Machuca, Alejandro Bruna-Mejias, Pablo Nova-Baeza, Juan José Valenzuela-Fuenzalida

**Affiliations:** a Escuela de Medicina, Universidad Finis Terrae, Santiago, Chile Departamento de Morfología, Facultad de Medicina; b Universidad Andres Bello, Santiago, Chile; c Department of Morphology and Function, Faculty of Health Sciences, Universidad de Las Américas, Santiago, Chile; d Departamento de Ciencias y Geografía, Facultad de Ciencias Naturales y Exactas, Universidad de Playa Ancha, Valparaíso, Chile; e Departamento de Ciencias Química y Biológicas, Facultad de Ciencias de la Salud, Universidad Bernardo O’Higgins, Santiago, Chile.

**Keywords:** alpha-lipoic acid, diabetic polyneuropathy, neuropathic pain, pharmacology, polyneuropathy, type I diabetes mellitus, type II diabetes mellitus

## Abstract

**Background:**

This systematic review explores the most current evidence regarding the mechanisms of neuropathic pain in patients with different types of diabetes and how this pain affects different functional and structural components of the neuroanatomical pain pathways. The review also seeks to provide guidelines for the best approach and treatment for patients experiencing this type of pain. The objective is to determine the effectiveness of alpha-lipoic acid (ALA) in improving functional and symptomatic outcomes in patients with diabetes mellitus type I and type II.

**Objective:**

To determine the effectiveness of alpha-lipoic acid (ALA) in improving functional and symptomatic outcomes in patients with diabetes mellitus type I and type II.

**Methods:**

We systematically search MEDLINE (via PubMed), EMBASE, SCOPUS, the Cochrane Central Register of Controlled Trials, the Cumulative Index to Nursing and Allied Health Literature, and Web of Science databases.

**Results:**

The findings of this review show that different forms of ALA do not present statistically significant changes for any of the scales included, including total symptom score (standardized mean difference [SMD] = −3.59, confidence interval [CI] = −4.16 to −3.02, and *P* < .00001), neuropathy impairment score (SMD = −1.42, CI = −3.68 to 0.84, and *P* = .22), and neuropathy symptom checklist (SMD = −0.09, CI = −0.15 to −0.02, and *P* = .01).

**Conclusion:**

In comparison to the use of a placebo, the findings suggest that ALA does not exhibit significant differences in terms of pain reduction and different functional scales. Moreover, no specific dosages are identified to support the use of ALA for the reduction of neuropathic pain.

## 1. Introduction

Diabetes mellitus (DM) is a chronic endocrine–metabolic disease characterized by a sustained rise in blood glucose caused by deficient insulin secretion or action and accompanied by changes in nutrient metabolism.^[1]^

There are multiple types of DM. Type I DM is characterized by a lack of insulin due to pancreatic β-cell dysfunction.^[2]^ Type 1 DM constitutes approximately 5% to 10% of all DM cases,^[1]^ and it is usually diagnosed in childhood.^[3]^ On the other hand, type II DM is characterized by persistent hyperglycemia despite the presence of hyperinsulinemia. Type II DM ranges from insulin resistance together with relative insulin deficiency to a progressive decline in insulin secretion. Type II DM is associated with obesity or increased visceral fat,^[4]^ and its diagnosis is made through routine examinations in asymptomatic patients.^[5]^ Type II DM is the most common type of diabetes, with an incidence of approximately 90% to 95% of all cases.^[1]^ DM is the leading cause of medical consultation for neuropathic pain (NP),^[6]^ and the prevalence of NP in patients with DM is estimated to be between 6% and 51% of cases.^[7]^

NP is defined as pain that is caused by an injury, disease, or dysfunction that affects the somatosensory system. This pain involves an alteration of the normal physiology of the neurons that integrate nociceptive information.^[8]^ Classification of neuropathies can be according to their location, distinguishing between central or peripheral, or according to their distribution, distinguishing between localized or diffuse.^[9]^ NP syndromes have a prevalence of 7% to 10% in the general population.^[10]^ Given that current treatments are not completely effective, it is necessary to seek alternative treatments with different mechanisms of action that are effective.^[11]^

Various studies have shown that hyperglycemia is a triggering factor in diabetic neuropathies. Therefore, the approach to addressing this problem should focus on this finding. Among the mechanisms that generate diabetic neuropathy, alterations in the metabolic pathways that produce oxidative stress due to the constant hypermetabolic state^[12]^ have been identified. Consequently, it is necessary to have in place effective antioxidant systems to prevent cell damage.

Alpha-lipoic acid (ALA) is considered a quintessential “universal antioxidant” due to its amphipathic nature and its powerful antioxidant power against free radicals.^[13]^ These properties make ALA a promising molecule in combating sensitization of the nervous system, such as in the case of diabetic neuropathies.

In addition to the low concentrations that the body produces, ALA is present in foods such as red meat, spinach, broccoli, wheat, and peas.^[14]^ This presence makes it feasible to use ALA supplementation for the treatment of oxidative stress associated with DM.^[15]^ One way to administer ALA is through the oral route, which is why ALA is marketed in the form of capsules. For the treatment of diabetic neuropathy associated with DM, the recommended dose is often 600 mg/day.^[16]^

Due to the reasons stated above, our main objective is to identify the effect of ALA in the treatment of peripheral neuropathy (PN) generated by DM. There is an urgent need to improve the quality of life of those affected by this condition through the administration of ALA treatment as a possible therapeutic approach to the management of the disease.

## 2. Methods

### 2.1. Literature search

This systematic review was conducted in accordance with the Preferred Reporting Items for Systematic Reviews and Meta-Analyses guidelines.^[18]^ This revision has been checked into the OSF repository with the following doi: 10.17605/OSF.IO/HMF7Y. We systematically searched electronic databases for the literature search, including MEDLINE (via PubMed), EMBASE, SCOPUS, the Cochrane Central Register of Controlled Trials, the Cumulative Index to Nursing and Allied Health Literature and Web of Science databases, covering records from the earliest time to May 2023. Randomized or controlled clinical trials that have been published in English or Spanish were included. The following keywords were used in different combinations: “diabetic neuropathy,” “ALA” and “neuropathic pain.” The search strategies for each database are available in the supplemental content (see Table S1, http://links.lww.com/MD/K338). Two authors (J.J.V.-F. and M.O.-D.) independently screened the titles and abstracts of the references retrieved from the searches. We obtained the full text for references that either author considered to be potentially relevant. We involved a third reviewer (P.N.-B.) if a consensus could not be reached.

### 2.2. Study selection

The inclusion criteria for the studies in this review were: patients with NP associated with type 1 or 2 DM; patients who were administered ALA in different doses and modes; reports of pain, disability and/or functionality; and studies that were aleatorized clinical trials, randomized clinical trials, and experimental studies. Studies were excluded if they: letters, case reports/series, reviews or non-human trials; studies that enrolled patients with other diseases; studies that administered other therapies in addition to ALA; or had no control group.

### 2.3. Data extraction and quality assessment

Two authors (M.B.-V. and M.L.-C.) independently extracted relevant data for each trial. The following data were extracted from the original reports: authors and year of publication; type of study and the total number of participants; outcome; statistical values and main results; geographical region; gender distribution; and doses of intervention and type of administration. Methodological quality of the included studies was evaluated by the Cochrane RoB tool.^[19]^ This tool assesses the RoB across 7 domains: generation of a random sequence, concealment of the randomization sequence, blinding of participants and treatments, blinding of the evaluation of the results, incomplete results, selective reporting of results, and other sources of bias. Each domain could be considered as having “low” RoB, “unclear” or “high” RoB.

Disagreements were resolved by discussion or determined by a third reviewer (J.J.V.-F.) if a consensus could not be reached. The agreement rate between the reviewers was calculated using kappa statistics, resulting in a substantial agreement with a value of 0.72.

### 2.4. Data synthesis and analysis

For the assessment of NP, various scales were used: total symptom score (TSS), neurological impairment scale (NIS), neuropathy symptoms and change score (NSC), and pain intensity. These scales were analyzed as continuous outcomes. The effect size was calculated as the standard mean difference (SMD). We calculated the SMD score using Cohen d as the effect size statistic, categorizing the effect sizes as trivial (<0.2), small (0.2–0.5), medium (0.6–0.8), or large (>0.8). Additionally, depending on the heterogeneity of the data, the Hartung–Knapp–Sidik–Jonkman random effect or Mantel–Haenszel fixed effect methods were used to quantify the pooled effect size of the studies included. We presented the effect sizes as SMD, with their respective 95% confidence intervals (CIs) in the range between 2 and −2. The heterogeneity of results across studies was evaluated using the I2 statistic, which considers 0% to 40% as “may not be important,” 30% to 60% as “moderate,” 50% to 90% as “substantial,” and 75% to 100% as “considerable” heterogeneity. Furthermore, we conducted a visual inspection to detect overlapping CIs in the forest plots as well as the corresponding *P* values. The meta-analysis was performed using RevMan 5.4.^[17,20]^

### 2.5. Rating the quality of evidence

The synthesis and quality of evidence for each outcome were assessed using the grading of recommendation, assessment, development and evaluation (GRADE).^[21]^ The quality of the evidence was classified into 4 categories: high, moderate, low and very low.^[22]^ We used the GRADE profiler to import the data from RevMan 5.4 to create a “summary of findings” table, which can be found in Supplementary Table 3, http://links.lww.com/MD/K340.

## 3. Results

### 3.1. Study selection

**Figure 1.**
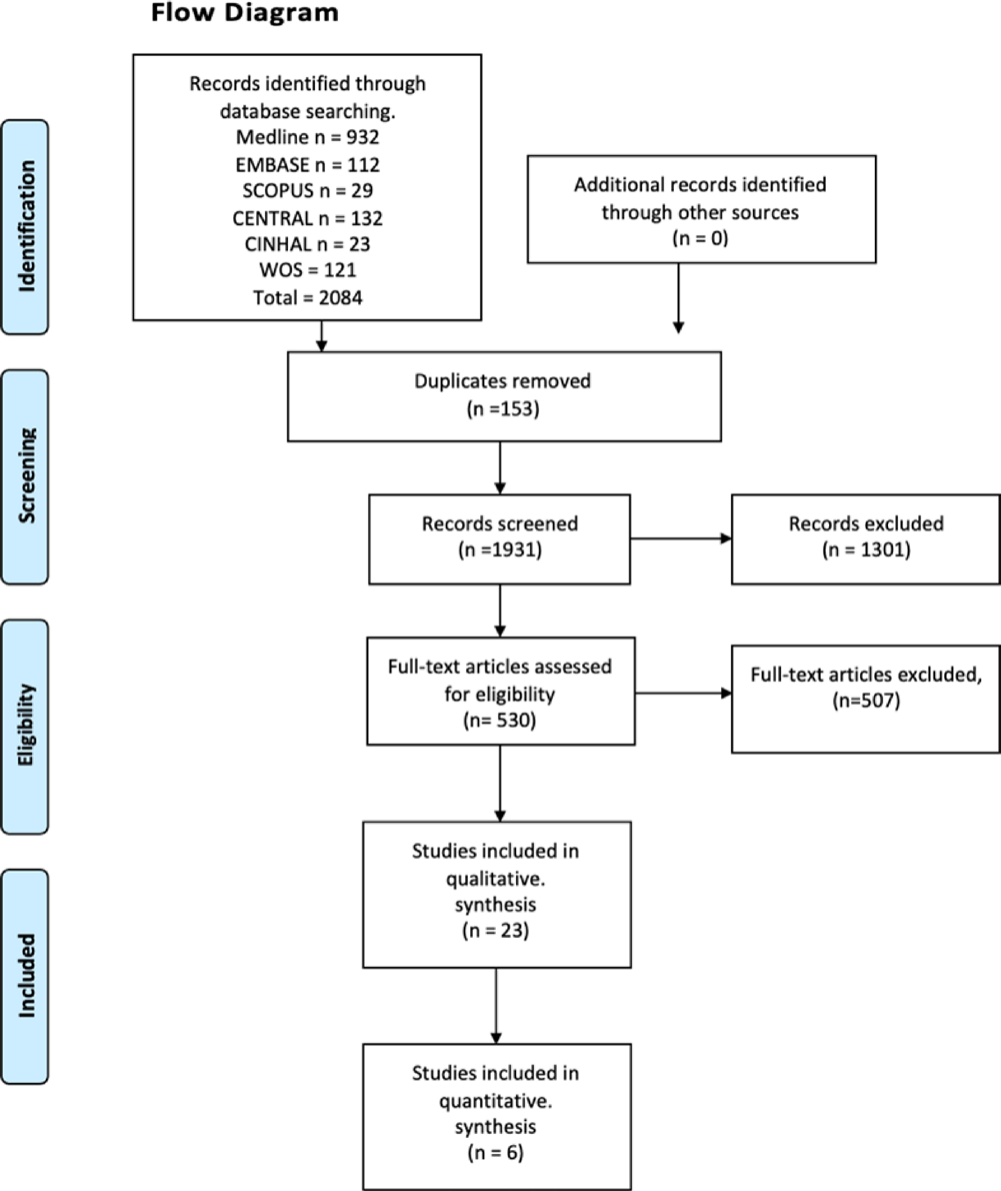
Flow diagram showing the study selection process based on the suggested format of Preferred Reporting Items for Systematic Reviews and Meta-Analyses guidelines.

A total 2084 of studies were found through electronic searches (Fig. 1). Ultimately, 6 trials met the eligibility criteria and were included in this systematic review.^[23–28]^ The kappa agreement rate between reviewers was 0.81. The excluded studies and the reasons for their exclusion are available in Table S2 of the supplemental content, http://links.lww.com/MD/K339.

### 3.2. Study characteristics

A summary of the included studies is presented in Table 1. The overall population included 1077 patients (661 in the ALA group and 416 in the placebo group). The mean age was 52.8 years (±2.1), and the mean follow-up duration was 31 days (ranging from 1–84).

**Table 1:** Summary of the characteristics of the studies included in the meta-analysis.

Author & yr	Type of study & N	Incidence and characteristics	Statistical values	Geography region	Gender	Doses
Ametov et al, 2003.	Clinical trial n = 120 2 patients in the placebo group withdrew from the study.	Metabolically stable DM patients with symptomatic sensorimotor polyneuropathy.	TSS decreased significantly −5.72 ± 1.53 points in the ALA group and 1.83 ± 1.97 points in the placebo group (*P* < .001). NSC had no significant differences between ALA and placebo groups. However, there was significant improvement in sensory neuropathic pain symptoms (*P* < .001 for most of them). NIS significantly decreased 2.7 ± 3.37 points in the ALA group and 1.2 ± 4.14 points in the placebo group (*P* < .001). NIS (LL) had no significant differences between the ALA and placebo groups (*P* = .076).	Russia	ALA: M = 14 F = 46 Placebo: M = 24 F = 36	Placebo for the entire sample for 1 wk. 600 mg ALA or placebo Monday through Friday for 2 wk and Monday through Thursday for 1 wk (14 total treatments). Intravenous administration for 3 wk.
Hahm et al, 2004	Clinical trial n = 38	DM patients with symptomatic polyneuropathy	TSS decreased significantly from 7.74 ± 2.22 to 3.4 ± 1.76 in the improved group and from 6.72 ± 3.42 to 6.63 ± 2.78 in the unchanged group (*P* = .005).	South Korea	Group with ALA 600 M = 13; F = 14 Placebo group M = 4 F = 7	ALA 600 mg/d Oral administration for 8 wk
Ruhnau et al, 1999	clinical trial n = 24 2 patients withdrew from the study, 1 ALA group and 1 Placebo group.	Type II DM patients with polyneuropathy.	TSS significantly decreased −3.75 ± 1.88 points in the ALA group and −1.94 ± 1.5 points in the placebo group (*P* = .021). HPAL decreased with borderline significance −2.20 ± 1.65 points in the ALA group and −0.96 ± 1.32 points in the placebo group (*P* = .072). NDS significantly decreased −0.27 ± 0.47 points in ALA group and increased + 0.18 ± 0.4 points in placebo group (*P* = .025).	Germany	ALA: M = 6 F = 6 Placebo: M = 6 F = 6	LA 600 mg 3 times a day Oral administration for 19 d.
Ziegler et al, 1995	Clinical trial n = 260	Non-insulin dependent DM patients with peripheral neuropathy	TSS was significantly decreased by −4.5 ± 3.7 points on ALA1200, −5.0 ± 4.1 points on ALA600, −3.3 ± 2.8 points on ALA100, and −2.6 ± 3.2 points on placebo. (ALA1200 vs Placebo: *P* = .003; ALA 600 vs Placebo: *P* < .001). There were no significant differences between ALA100 and placebo. HPAL significantly decreased for ALA1200 and ALA600 vs placebo (both *P* < .01). There were no significant differences between ALA100 and placebo. NDS decreased −1.8 ± 0.3 points on ALA1200, −1.5 ± 0.3 points on ALA600, −0.9 ± 0.3 on ALA100, and −1.0 ± 0.2 on placebo. (ALA1200 vs Placebo: *P* = .03)	Germany	ALA1200: M = 26 F = 39 ALA600: M = 23 F = 40 ALA100: M = 34 F = 32 Placebo: M = 23 F = 43	ALA1200: 1200 mg/d for 2 periods of 5 d for 3 wk ALA600: 600 mg/d of ALA ALA100: 100 mg/d Placebo: 250 mg/d Intravenous administration for 19 d.
Ziegler et al, 2006	Clinical trial n = 181	DM patients with distal symmetric polyneuropathy with positive sensory symptoms and neuropathic deficits	TSS was significantly decreased by −4.85 ± 3.03 points in ALA600, −4.5 ± 3.28 points in ALA1200, −4.7 ± 3.54 points in ALA1800, and −2.92 ± 3.18 points in placebo (*P* < .05). NSC decreased significantly −2.8 ± 2.1 points in ALA600 and −2.8 ± 2.2 points in ALA1200 (ALA600 and ALA1200 vs Placebo: *P* < .05). NSC decreased with borderline significance −2.7 ± 2.5 points in ALA1800 and −1.7 ± 2.1 points in placebo (*P* = .08). NIS (LL) decreased with borderline significance −3.75 ± 4.41 points in ALA600, −2.63 ± 3.28 points in ALA1200, −2.7 ± 5.33 points in ALA1800 and −2.08 ± 5.57 in placebo. (ALA600 vs Placebo: *P* = .07; ALA1200 vs Placebo: *P* = .09).	Russian	Placebo: M = 15 F = 28 ALA600: M = 20 F = 25 ALA1200: M = 19 F = 28 ALA1800: M = 19 F = 27	ALA600: 600 mg/d of ALA ALA1200: 1200 mg/d of ALA ALA1800: 1800 mg/d of ALA Placebo Oral administration for 5 wk, after 1 wk of placebo
Ziegler et al, 2011	Clinical trial n = 454	DM patients with mild to moderate diabetic symmetric distal sensorimotor polyneuropathy	TSS decreased −0.22 ± 2.42 points in the ALA group and −0.21 ± 2.45 points in the placebo group. NSC significantly decreased −0.04 ± 0.26 points in ALA group and increased + 0.04 ± 0.42 points in placebo group (*P* = .005 and *P* = .008 respectively). NIS significantly decreased −0.68 ± 6.44 points in the ALA group and increased + 0.61 ± 6.61 points in the placebo group (*P* = .028). NIS(LL) significantly decreased −0.34 ± 4.48 points in the ALA group and increased + 0.43 ± 4.49 points in the placebo group (*P* = .045).	Germany	ALA M = 152 F = 78 Placebo M = 150 F = 74	600 mg/d of ALA or Placebo Oral administration for 4 yr

ALA = alpha-lipoic acid, DM = diabetes mellitus, HPAL = Hamburg pain adjective list, NDS: neuropathy disability score, NIS = neurological impairment scale, NIS (LL) = neuropathy impairment score in the lower limbs, NSC = neuropathy symptoms and change score, NSS = neuropathy symptoms score, TSS = total symptom score.

### 3.3. Risk of bias assessment in individual studies

**Figure 2.**
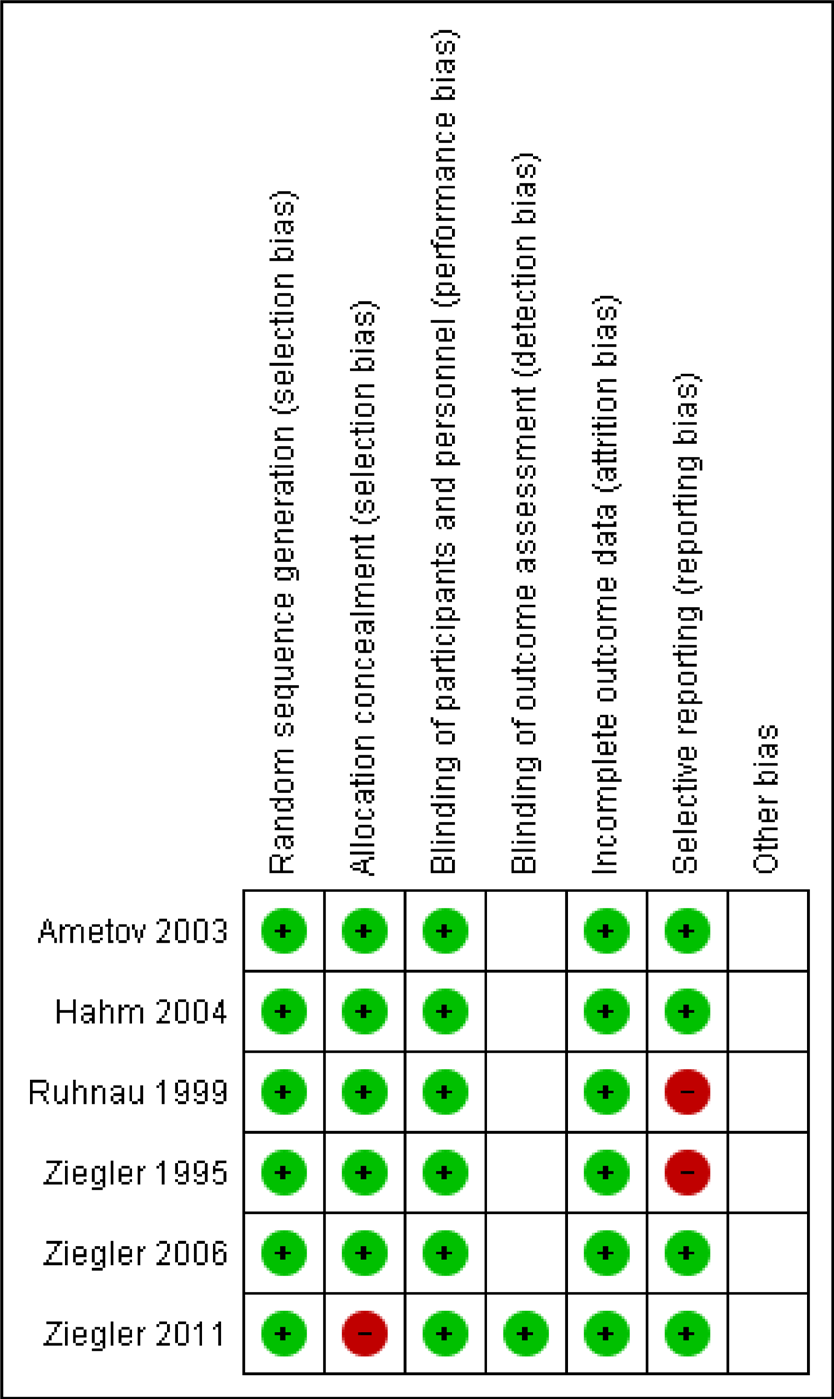
Risk of bias summary: review authors judgements about each risk of bias item for each included study.

**Figure 3.**
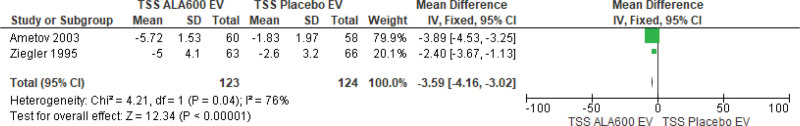
Forest plot of comparisons of the total symptom score (TSS) standardized mean difference (SMD) between ALA600 EV and Placebo.

The RoB2 assessment is presented in Figures 2 and 3. Regarding overall bias, 50% of the studies were classified as having “some concerns,”^[25–27]^ while the remaining 50% were classified as having a “low risk” of bias.^[23,27]^ For the randomization process, 100% of the trials received a “low risk” of bias rating.^[23–28]^ For the incomplete outcome data, 100% of the trials were scored as “low risk.”^[23–28]^ Finally, for the selection of the reported result, 33.3% of the trials were scored as “high risk”^[25,26]^ and 66.6% were scored as having a “low risk” of bias^[23,24,27,28]^ (see Fig. 2).

### 3.4. Synthesis of results

Regarding the studies that exhibited some homogeneity of treatment and evaluation, we included 6 studies in this meta-analysis. The evaluation scales employed in these studies were TSS, NSC, and NIS. The administration and doses of ALA were done through oral and intravenous (IV) routes, with doses of 600 mg/day and 1800 mg/day. The results of each evaluation scale are detailed below.

#### 3.4.1. TSS.

##### 3.4.1.1. TSS ALA600 IV.

Two studies provided data used to perform a meta-analysis to assess associated symptoms in patients with NP and DM using the TSS scale.^[23,26]^ These studies showed no significant difference in the pooled SMD estimate between ALA600 IV versus a placebo IV (SMD = −3.59, CI = −4.16 to −3.02, and *P* < .00001), with a substantial heterogeneity (I^2^ = 76% and *P* = .04).^[23,26]^ These results are presented in Figure 3. The quality of evidence, based on the GRADE rating, was determined to be very low.

##### 3.4.1.2. TSS ALA600 ORAL.

**Figure 4.**
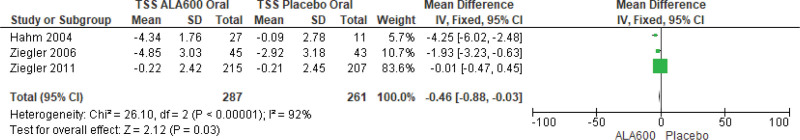
Forest plot of comparisons of the total symptom score (TSS) standardized mean difference (SMD) between ALA600 Oral and Placebo.

Three studies provided data used to perform a meta-analysis using the TSS scale to assess associated symptoms in patients with NP and DM.^[24,27,28]^ These studies showed no significant difference in the pooled SMD estimate between ALA600 ORAL and the oral placebo (SMD = −0.46, CI = −0.88 to −0.03, and *P* = .03) and had substantial heterogeneity (I^2^ = 92% and *P* < .00001).^[24,27,28]^ These results are presented in Figure 4. The quality of evidence, based on the GRADE rating, was determined to be very low.

##### 3.4.1.3. TSS ALA1800 ORAL.

**Figure 5.**
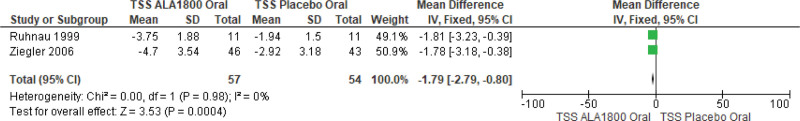
Forest plot of comparisons of the total symptom score (TSS) standardized mean difference (SMD) between ALA1800 Oral and Placebo.

Two studies included data used to perform a meta-analysis to assess associated symptoms in patients with NP and DM using the TSS scale.^[25,27]^ These studies showed no significant difference in the pooled SMD estimate between oral ALA1800 versus an oral placebo (SMD = −1.79, CI = −2.79 to −0.80, and *P* = .0004) and had a substantial heterogeneity (I^2^ = 0% and *P* = .98).^[25,27]^ These results are presented in Figure 5. There was a very low quality of evidence according to the GRADE rating.

#### 3.4.2. NSC ALA600 ORAL.

**Figure 6.**
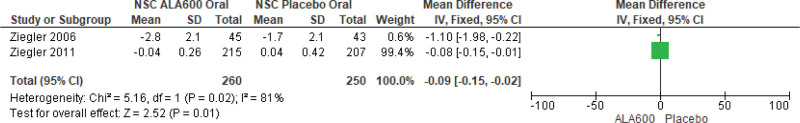
Forest plot of comparisons of the neuropathy symptoms and change score (NSC) standardized mean difference (SMD) between ALA600 Oral and Placebo.

Two studies included data used to perform a meta-analysis to assess associated symptoms in patients with NP and DM using the NSC scale.^[27,28]^ These studies showed no significant difference in the pooled SMD estimate between oral ALA600 versus an oral placebo (SMD = −0.09, CI = −0.15 to −0.02, and *P* = .01) and had a substantial heterogeneity (I^2^ = 81% and *P* = .02).^[27,28]^ These results are presented in Figure 6. There was a very low quality of evidence according to the GRADE rating.

#### 3.4.3. NIS ALA600 ORAL.

**Figure 7.**
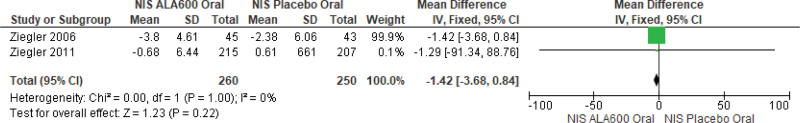
Forest plot of comparisons of the NIS standardized mean difference (SMD) between ALA600 Oral and Placebo.

Two studies included data used to perform a meta-analysis to assess associated symptoms in patients with NP and DM using the NIS scale.^[27,28]^ These studies showed no significant difference in the pooled SMD estimate between oral ALA600 versus an oral placebo (SMD = −1.42, CI = −3.68 to 0.84, and *P* = .22), with a substantial heterogeneity (I^2^ = 0 and *P* = 1.00).^[27,28]^ These results are presented in Figure 7. There was a low quality of evidence according to the GRADE rating.

## 4. Discussion

This systematic review and meta-analysis aimed to determine the clinical effectiveness of ALA in patients with neuropathic pain associated with DM type 1 and 2. The main findings of our study are that ALA, at different doses and through different modes of administration, does not provide a significant benefit in terms of symptomatological reduction of NP across different assessment scales when compared to a placebo.

Regarding previous meta-analyses or systematic reviews that have investigated the effect of ALA in patients with diabetic neuropathy associated with DM, we found 4 articles detailing the aforementioned parameters. In the review by Ziegler 1997,^[29]^ where he evaluated the use of ALA in patients with peripheral polyneuropathy associated with DM, the main result was that the administration of 600 mg/day of ALA through the IV route for 3 weeks is safe and effective. Additionally, when it comes to reducing symptoms of diabetic PN, the administration of 800 mg/day of ALA orally for 4 months can improve cardiac autonomic dysfunction in patients with non-insulin-dependent DM. The above findings differ from the results of our study in that we believe that there is no specific dose that presents better results than another. Neither can we support a specific route of administration, and the efficacy time is also questionable. Finally, our study differs from that of Ziegler (1997) in that the focus of our meta-analysis was a comparison of pain outcomes,^[29]^ which showed no substantive evidence supporting the use of ALA.

Regarding the review by Tang 2007,^[30]^ the main results were based on a randomized clinical trial showing a decrease in distal symptoms of diabetic polyneuropathy, mainly based on decreased pain, burning, paresthesia, and distal numbness associated with diabetic foot. In comparison, our study differed from that of Tang since we were able to compare outcomes from different studies, thus providing a comprehensive perspective on the need of additional evidence to support the recommendation of ALA as a gold standard for patients with diabetic polyneuropathy.

The review by Nguyen 2018^[31]^ demonstrated that the use of ALA is efficacious in the treatment of PN associated with DM. However, our study specifies different doses associated with different administrations and with specific pain and functionality scales. We found no solid evidence to propose the exclusive use of ALA.

Finally, the review by Jiang 2018^[32]^ proposed that the administration of ALA combined with epalrestat is an effective option for patients with PN associated with DM. Jiang performed an exhaustive meta-analysis measuring different outcomes resulting from the combination of ALA with another drug. However, his analysis differs from our study because we only tried to demonstrate the efficacy of the use of ALA for PN when compared to other pharmacological modalities of intervention.

Regarding NP diagnosis, the International Association for the Study of Pain defines NP as pain arising as a direct consequence of injury or a disease affecting the somatosensory system. In 2011, the International Association for the Study of Pain Neuropathic Pain Special Interest Group proposed a classification system for NP, categorizing it as definite, probable, and unconfirmed. If the pain distribution is not neuroanatomically plausible and diagnostic testing (MRI and/or electroneuromyography) does not show peripheral nerve injury or central nervous system injury, the pain is considered unconfirmed as NP.^[29]^ This discrepancy in the definition of NP means that its diagnosis may be deficient because it may be either overestimated or underestimated by the patient. This discrepancy is accentuated since there are no definitions or classifications that support the diagnosis of NP in different regions of the body. Nevertheless, all trials included in this systematic review recruited patients with a clinical diagnosis of DM with symptomatic polyneuropathy lasting over a year.^[23,25–28]^ Only one trial included patients with a diagnostic evolution ranging from months to years.^[24]^

In terms of the analyzed evidence, there is a high degree of heterogeneity in the doses and types of ALA administration used for patients with NP associated with diabetes. Two clinical trials studied 600 mg of IV ALA, 3 studied 600 mg of oral ALA, and 2 studied 1800 mg of oral ALA.^[23–28]^

The application of ALA, as mentioned, was explored using different doses and routes of administration. Furthermore, the period of treatment ranged between 18 months and 4 years, with a periodicity that varied between 2 and 3 days per week. In patients with PN associated with DM, the central and peripheral mechanisms are highly involved in the appearance of these symptoms for which several therapeutic alternatives have been proposed. Pharmacological treatments vary greatly, and many drugs have been proposed. However, the evidence for these treatments is meager.

One of the suggested pharmacological treatments is the use of ALA, which has proven to be beneficial for treating symptomatic diabetic polyneuropathy. Studies have observed a clear decrease in symptoms after 5 weeks of continuous use. However, the analgesic effect decreases after the third week following the end of treatment. A daily oral dose of 600 mg/day is suggested, since it improves pain and is well tolerated by the patient. The most frequently observed side effects in the majority of patients are nausea, vomiting, and dizziness (Ziegler 2006).^[27]^

Based on our findings, ALA was studied in various doses and through different routes of administration. ALA does not have an established dose, and no dosage has been shown to be better than another. Similarly, there is no solid evidence regarding the superiority of one route of administration over another. Finally, in the reviewed studies,^[23–28]^ there was no significant difference between ALA and other drugs: there were no differences in clinical benefits.

ALA is currently recognized for its antioxidant properties and its potential in managing central sensitization pathologies and chronic pain.^[33,34]^ ALA acts as a powerful neutralizer of free radicals and metal chelate-formers in addition to having opioid effects associated with decreased sensitization of the central nervous system and peripheral components underlying NP. Studies have demonstrated these effects using an approximate dose of 10 mg ALA/kg/ 30 days. In the present review, we did not find studies that provide evidence that at the end of treatment, the beneficial effects continue or are maintained in patients with NP.^[35,36]^

The clinical implications of our findings are limited by the quality and quantity of the available evidence. The use of different proposed doses and the different types of administration led to a high degree of heterogeneity in the samples, and not all of the samples could be directly compared. Moderate-quality evidence suggests that the use of ALA is beneficial for the treatment of PN associated with DM: the gold standard dose is 600 mg/day. Based on our findings, it is not possible to make clinical recommendations on the use of ALA in patients with NP and DM because although ALA could be useful in relieving pain, improving function, or reducing disability, there is no consensus regarding dosage, routes of administration, and treatment duration. At the same time, studies show that stopping the use of ALA indefinitely or permanently could have non-beneficial side effects for patients.

Some limitations in our study must be recognized. First, even though we searched 6 databases and included papers from 2 different languages, we may have missed articles relevant to our search. Second, in the planning stages, we proposed to perform subgroup analyses based on dose and route of administration. However, it was difficult to perform the analyses exhaustively due to the high heterogeneity of the groups and clinical signs among the included studies. Third, the lack of adequate sample sizes, unclear allocation concealment, and a lack of blinding of patients and assessors in the included studies could have led to an overestimation of the effect size of the interventions studied. Finally, our results must be interpreted with caution in relation to methodological limitations, the high heterogeneity of the included studies, and the limited strength of the available evidence.

## 5. Limitations

This review has limitations. Firstly, the included studies have a publication bias: studies with different results that were in non-indexed literature in the selected databases may have been left out. Secondly, there is a probability that a most sensitive and specific search regarding the topic to be studied was not carried out. Finally, personal preference may have influenced the authors in the selection of articles.

## 6. Conclusion

The use of ALA compared to the use of a placebo did not lead to significant differences in terms of pain reduction and different functional scales. In addition, we did not find doses that can support the use of ALA for the reduction of PN. It is important to note that based on GRADE analysis, the evidence in favor of or against the use of ALA in patients with diabetes is low to moderate, and additional high quality studies with a large number of patients are NOT needed.

## Author contributions

**Conceptualization:** Juan José Valenzuela-Fuenzalida, Michelle Lopez-Chaparro, Marisol Barahona-Vasquez, Alejandro Bruna-Mejias.

**Data curation:** Juan José Valenzuela-Fuenzalida, Michelle Lopez-Chaparro, Mathias Orellana-Donoso.

**Formal analysis:** Mathias Orellana-Donoso.

**Investigation:** Juan José Valenzuela-Fuenzalida, Marisol Barahona-Vasquez.

**Methodology:** Juan José Valenzuela-Fuenzalida, Michelle Lopez-Chaparro, Marisol Barahona-Vasquez.

**Project administration:** Juan José Valenzuela-Fuenzalida, Marisol Barahona-Vasquez, Alejandro Bruna-Mejias, Pablo Nova-Baeza.

**Resources:** Alejandro Bruna-Mejias, Pablo Nova-Baeza.

**Supervision:** Juan José Valenzuela-Fuenzalida, Michelle Lopez-Chaparro.

**Software:** Marisol Barahona-Vasquez, Alejandro Bruna-Mejias.

**Validation:** Juan José Valenzuela-Fuenzalida, Andres Santana-Machuca, Pablo Nova-Baeza.

**Visualization:** Juan José Valenzuela-Fuenzalida, Andres Santana-Machuca, Mathias Orellana-Donoso.

**Writing – original draft:** Juan José Valenzuela-Fuenzalida, Michelle Lopez-Chaparro, Andres Santana-Machuca, Mathias Orellana-Donoso, Pablo Nova-Baeza

**Writing – review & editing:** Juan José Valenzuela-Fuenzalida, Michelle Lopez-Chaparro, Marisol Barahona-Vasquez, Andres Santana-Machuca, Mathias Orellana-Donoso, Pablo Nova-Baeza.

## Supplementary Material
