# Supplementary material for: Effectiveness of alpha-lipoic acid in patients with neuropathic pain associated with type I and type II diabetes mellitus: A systematic review and meta-analysis
Source: Medicine (Baltimore). 2023 Nov 3;102(44):e35368. doi: 10.1097/MD.0000000000035368 (PMC10627688; doi:10.1097/MD.0000000000035368)
Supplement: Supplementary file 1 [file medi-102-e35368-s001.docx]

**A<Supplemental Digital Content. Table S1.** Searches strategies

| Database | Search strategy | Results | |
| --- | --- | --- | --- |
|  |  | 27-05-23 |  |
| Medline | ((((((((Acid alpha lipoic) OR (Pharmacology)) AND (type I diabetes mellitus)) OR (type II diabetes mellitus)) AND (Neuropathic pain)) OR (diabetic polyneuropathy)) OR (polyneuropathy)) AND (randomized clinical trial)) NOT (animals) Filters: Clinical Trial | 1667 |  |
| EMBASE | Acid alpha lipoic OR (AND (type I diabetes mellitus)) OR (type II diabetes mellitus)) AND (Neuropathic pain)) OR (diabetic polyneuropathy)) OR (polyneuropathy)) AND (randomized clinical trial)) | 112 |  |
| SCOPUS | (Acid alpha lipoic) OR (Pharmacology)) AND (type I diabetes mellitus)) OR (type II diabetes mellitus)) AND (Neuropathic pain)) OR (diabetic polyneuropathy)) OR (polyneuropathy)) AND (randomized clinical trial)) | 29 |  |
| CENTRAL | (Acid alpha lipoic) OR (Pharmacology)) AND (type I diabetes mellitus)) OR (type II diabetes mellitus)) AND (Neuropathic pain)) OR (diabetic polyneuropathy)) OR (polyneuropathy)) AND (randomized clinical trial)) | 132 |  |
| CINHAL | Acid alpha lipoic) AND (type I diabetes mellitus)) OR (type II diabetes mellitus)) AND (Neuropathic pain)) OR (diabetic polyneuropathy)) OR (polyneuropathy)) AND (randomized clinical trial)) | 23 |  |
| WOS | Acid alpha lipoic) OR (Pharmacology)) AND (type I diabetes mellitus)) OR (type II diabetes mellitus)) AND (Neuropathic pain)) OR (diabetic polyneuropathy)) OR (polyneuropathy)) AND (randomized clinical trial | 121 |  |
|  | Total | 2084 |  |

* All searches were carried out on May 27, 2023.
