# Supplementary material for: Effectiveness of alpha-lipoic acid in patients with neuropathic pain associated with type I and type II diabetes mellitus: A systematic review and meta-analysis
Source: Medicine (Baltimore). 2023 Nov 3;102(44):e35368. doi: 10.1097/MD.0000000000035368 (PMC10627688; doi:10.1097/MD.0000000000035368)
Supplement: Supplementary file 2 [file medi-102-e35368-s002.docx]

**Supplemental Digital Content. Table S3.** Summary of Findings (SoF) and quality of evidence (GRADE) for Acid alpha lipoic in patient’s whit neuropathic pain associated diabetes mellitus.

| **Certainty assessment** | | | | | | | **№ of patients** | | **Effect** | | **Quality of evidence (GRADE)** | **Importance** |
| --- | --- | --- | --- | --- | --- | --- | --- | --- | --- | --- | --- | --- |
| **№ of studies** | **Study design** | **Risk of bias** | **Inconsistency** | **Indirectness** | **Imprecision** | **Other considerations** | **ALA Modalities dose and administration** | **Placebo** | **Relative (95% CI)** | **SMD (95% CI)** |  |  |
| **TSS ALA600 EV (Follow-up: 2 to 18 weeks)** | | | | | | | | | | | | |
| 2 | RCT | Serious | Very serious | Not serious | Serious | None | 123 | 124 | - | SMD -3.59, (4.16 to -3.02) | ⨁◯◯◯ Very low | CRITCAL |
| **TSS ALA600 Oral (Follow-up: 2 weeks to 4 years)** | | | | | | | | | | | | |
| 3 | RCT | Serious | Very serious | Not serious | Serious | None | 287 | 261 | - | SMD -0.46 (-0.88 to -0.03) | ⨁◯◯◯ Very low | CRITICAL |
| **TSS ALA1800 Oral (Follow-up: 2 to 18 weeks)** | | | | | | | | | | | | |
| 2 | RCT | Not serious | Very serious | Not serious | Serious | None | 57 | 54 | - | SMD -1.79 (2.79 -0.80) | ⨁◯◯◯ Very low | CRITICAL |
| **NSS ALA 600 Oral (follow up: 2 weeks to 4 years)** | | | | | | | | | | | | |
| 2 | RCT | Serious | Very serious | Not serious | Serious | None | 260 | 250 | - | SMD -0.09 (-0.15 to -0.02) | ⨁◯◯◯ Very low | IMPORTANT |

**NiS ALA 600 Oral (follow up: 2 weeks to 4 years)**

| 2 | RCT | Serious | Very serious | Not serious | Serious | None | 260 | 250 | - | SMD = 1.42  (3.68 to 0.84) | ⨁⨁◯◯ Low | IMPORTANT |
| --- | --- | --- | --- | --- | --- | --- | --- | --- | --- | --- | --- | --- |

**SMD:** Standard Mean Difference; **RCT:** Randomized clinical trial; **Quality of evidence:** High: The research provides a very good indication of the likely effect. The probability that the effect is different is low; Moderate: The research provides a good indication of the likely effect. The probability that the effect is substantially different is moderate; Low: The research gives some indication of the probable effect. However, the probability that the effect is substantially different is high; Very low: The research does not provide a reliable estimate of the probable effect. The probability that the effect is substantially different is very high. **Downgrading:** GRADE approach has four reasons for possible rate down the quality of evidence. Begins with the study designs (trials or observational studies), secondly downgrading the evidence one level: (1) for study limitation if the majority of studies (>50%) was rated as high risk of bias; (2) for inconsistency, if heterogeneity was greater than the accepted low level (I^2^ >40%); (3) for indirectness, directness was undoubled; (4) for imprecision, if meta-analysis had a small sample size (n <400) or confidence interval very wide.
