# Supplementary material for: Effectiveness of alpha-lipoic acid in patients with neuropathic pain associated with type I and type II diabetes mellitus: A systematic review and meta-analysis
Source: Medicine (Baltimore). 2023 Nov 3;102(44):e35368. doi: 10.1097/MD.0000000000035368 (PMC10627688; doi:10.1097/MD.0000000000035368)
Supplement: Supplementary file 3 [file medi-102-e35368-s003.docx]

Table supplementary 2

**Supplemental Digital Content. Table S2.** Excluded studies and reasons.

|  | **Reference** | **Reason** |
| --- | --- | --- |
| 1 | Bertolotto F, Massone A. Combination of alpha lipoic acid and superoxide dismutase leads to physiological and symptomatic improvements in diabetic neuropathy. Drugs R D. 2012 Mar 1;12(1):29-34. doi: 10.2165/11599200-000000000-00000. PMID: 22329607; PMCID: PMC3586118. | Additional treatments evaluated. |
| 2 | Bureković A, Terzić M, Alajbegović S, Vukojević Z, Hadzić N. The role of alpha-lipoic acid in diabetic polyneuropathy treatment. Bosn J Basic Med Sci. 2008 Nov;8(4):341-5. doi: 10.17305/bjbms.2008.2894. PMID: 19125705; PMCID: PMC5677278. | Unmatched pain scale. |
| 3 | Didangelos T, Karlafti E, Kotzakioulafi E, Kontoninas Z, Margaritidis C, Giannoulaki P, Kantartzis K. Efficacy and Safety of the Combination of Superoxide Dismutase, Alpha Lipoic Acid, Vitamin B12, and Carnitine for 12 Months in Patients with Diabetic Neuropathy. Nutrients. 2020 Oct 23;12(11):3254. doi: 10.3390/nu12113254. PMID: 33114210; PMCID: PMC7690794. | Additional treatments evaluated. |
| 4 | Esposito C, Ugo Garzarella E, Santarcangelo C, Di Minno A, Dacrema M, Sacchi R, Piccinocchi G, Piccinocchi R, Daglia M. Safety and efficacy of alpha-lipoic acid oral supplementation in the reduction of pain with unknown etiology: A monocentric, randomized, double-blind, placebo-controlled clinical trial. Biomed Pharmacother. 2021 Dec;144:112308. doi: 10.1016/j.biopha.2021.112308. Epub 2021 Oct 12. PMID: 34649217. | Administered dose unmatched. |
| 5 | Garcia-Alcala H, Santos Vichido CI, Islas Macedo S, Genestier-Tamborero CN, Minutti-Palacios M, Hirales Tamez O, García C, Ziegler D. Treatment with α-Lipoic Acid over 16 Weeks in Type 2 Diabetic Patients with Symptomatic Polyneuropathy Who Responded to Initial 4-Week High-Dose Loading. J Diabetes Res. 2015;2015:189857. doi: 10.1155/2015/189857. Epub 2015 Aug 4. PMID: 26345602; PMCID: PMC4539458. | No comparison group. |
| 6 | Haak ES, Usadel KH, Kohleisen M, Yilmaz A, Kusterer K, Haak T. The effect of alpha-lipoic acid on the neurovascular reflex arc in patients with diabetic neuropathy assessed by capillary microscopy. Microvasc Res. 1999 Jul;58(1):28-34. doi: 10.1006/mvre.1999.2151. PMID: 10388600. | No comparison group. |
| 7 | Huang EA, Gitelman SE. The effect of oral alpha-lipoic acid on oxidative stress in adolescents with type 1 diabetes mellitus. Pediatr Diabetes. 2008 Jun;9(3 Pt 2):69-73. doi: 10.1111/j.1399-5448.2007.00342.x. Epub 2008 Jan 22. PMID: 18221433. | No pain scales are evaluated. |
| 8 | Jin HY, Joung SJ, Park JH, Baek HS, Park TS. The effect of alpha-lipoic acid on symptoms and skin blood flow in diabetic neuropathy. Diabet Med. 2007 Sep;24(9):1034-8. doi: 10.1111/j.1464-5491.2007.02179.x. Epub 2007 May 8. PMID: 17490418. | No reported results for the placebo group. |
| 9 | Konrad T, Vicini P, Kusterer K, Höflich A, Assadkhani A, Böhles HJ, Sewell A, Tritschler HJ, Cobelli C, Usadel KH. alpha-Lipoic acid treatment decreases serum lactate and pyruvate concentrations and improves glucose effectiveness in lean and obese patients with type 2 diabetes. Diabetes Care. 1999 Feb;22(2):280-7. doi: 10.2337/diacare.22.2.280. PMID: 10333946. | No pain scales are evaluated. |
| 10 | Li N, Yan W, Hu X, Huang Y, Wang F, Zhang W, Wang Q, Wang X, Sun K. Effects of oral α-lipoic acid administration on body weight in overweight or obese subjects: a crossover randomized, double-blind, placebo-controlled trial. Clin Endocrinol (Oxf). 2017 May;86(5):680-687. doi: 10.1111/cen.13303. Epub 2017 Feb 26. PMID: 28239907. | No pain scales are evaluated. |
| 11 | Nádró B, Lőrincz H, Molnár Á, Szentpéteri A, Zöld E, Seres I, Páll D, Paragh G, Kempler P, Harangi M, Sztanek F. Effects of alpha-lipoic acid treatment on serum progranulin levels and inflammatory markers in diabetic neuropathy. J Int Med Res. 2021 May;49(5):3000605211012213. doi: 10.1177/03000605211012213. PMID: 34041950; PMCID: PMC8165837. | No pain scales are evaluated. |
| 12 | Pieralice S, Vari R, Minutolo A, Maurizi AR, Fioriti E, Napoli N, Pozzilli P, Manfrini S, Maddaloni E. Biomarkers of response to alpha-lipoic acid ± palmitoiletanolamide treatment in patients with diabetes and symptoms of peripheral neuropathy. Endocrine. 2019 Nov;66(2):178-184. doi: 10.1007/s12020-019-01917-w. Epub 2019 Apr 4. PMID: 30949911. | Additional treatments evaluated. |
| 13 | Reljanovic M, Reichel G, Rett K, Lobisch M, Schuette K, Möller W, Tritschler HJ, Mehnert H. Treatment of diabetic polyneuropathy with the antioxidant thioctic acid (alpha-lipoic acid): a two year multicenter randomized double-blind placebo-controlled trial (ALADIN II). Alpha Lipoic Acid in Diabetic Neuropathy. Free Radic Res. 1999 Sep;31(3):171-9. doi: 10.1080/10715769900300721. PMID: 10499773. | No pain scales are evaluated. |
| 14 | Ruessmann HJ; German Society of out patient diabetes centres AND (Arbeitsgemeinschaft niedergelassener diabetologisch tätiger Arzte e.V.). Switching from pathogenetic treatment with alpha-lipoic acid to gabapentin and other analgesics in painful diabetic neuropathy: a real-world study in outpatients. J Diabetes Complications. 2009 May-Jun;23(3):174-7. doi: 10.1016/j.jdiacomp.2008.02.002. Epub 2008 Apr 9. PMID: 18403218. | Additional treatments evaluated. |
| 15 | Tankova T, Cherninkova S, Koev D. Treatment for diabetic mononeuropathy with alpha-lipoic acid. Int J Clin Pract. 2005 Jun;59(6):645-50. doi: 10.1111/j.1742-1241.2005.00452.x. PMID: 15924591. | Administered dose unmatched. |
| 16 | Ziegler D, Hanefeld M, Ruhnau KJ, Hasche H, Lobisch M, Schütte K, Kerum G, Malessa R. Treatment of symptomatic diabetic polyneuropathy with the antioxidant alpha-lipoic acid: a 7-month multicenter randomized controlled trial (ALADIN III Study). ALADIN III Study Group. Alpha-Lipoic Acid in Diabetic Neuropathy. Diabetes Care. 1999 Aug;22(8):1296-301. doi: 10.2337/diacare.22.8.1296. PMID: 10480774. | Administered dose unmatched. |
| 17 | Ziegler D, Low PA, Freeman R, Tritschler H, Vinik AI. Predictors of improvement and progression of diabetic polyneuropathy following treatment with α-lipoic acid for 4 years in the NATHAN 1 trial. J Diabetes Complications. 2016 Mar;30(2):350-6. doi: 10.1016/j.jdiacomp.2015.10.018. Epub 2015 Nov 10. PMID: 26651260. | Duplicated information. |
